# Making the Move: A Mixed Research Integrative Review

**DOI:** 10.3390/healthcare3030757

**Published:** 2015-08-26

**Authors:** Sarah Gilbert, Elaine Amella, Barbara Edlund, Lynne Nemeth

**Affiliations:** 1 Radford University, P.O. Box 6964 Radford, VA 24141, USA; 2 Medical University of South Carolina, 99 Jonathan Lucas Street MSC 160, Charleston, SC 29425, USA; E-Mails: amellaej@musc.edu (E.A.); edlundb@musc.edu (B.E.); nemethl@musc.edu (L.N.)

**Keywords:** relocation, transition, relocation, older adults, elderly, Theory of Planned Behavior, integrative review, adult children, supervised housing

## Abstract

The purpose of this mixed research integrative review is to determine factors that influence relocation transitions for older adults who are considering a move from independent living to supervised housing, such as assisted living, using the Theory of Planned Behavior as a conceptual guide. PubMED, CINAHL, and PsychInfo databases were queried using key words: relocation, transition, older adults, and, elderly and time limited from 1992 to 2014. Sixteen articles were retained for review. The majority of articles, qualitative in design, reveal that older adults who comprehend the need to move and participate in the decision-making process of a relocation adjust to new living environments with fewer negative outcomes than older adults who experience a forced relocation. The few quantitative articles examined the elements of impending relocation using a variety of instruments but support the necessity for older adults to recognize the possibility of a future move and contribute to the relocation process. Additionally, the influence of family, friends, and health care providers provides the older adult with support and guidance throughout the process.

## 1. Introduction

The aging demographic is exploding. Globally, the number of people aged 60 and over will triple from 605 million in 2000 to 2 billion in 2050 [1]. The number of older Americans, aged 65 and over will double from 2000 to 2040 and, by 2040, older adults will comprise 21% of the U.S. population [2]. This aging explosion presents challenges to society, governments, and families; however the greatest challenge will be for the older adult who must adapt to changes in physical functioning, the struggle to control chronic illnesses, and perhaps face the daunting challenge of a relocation and transition to a less independent living arrangement. Defined by Meleis [3] transition is a change or move from one place to another or from one condition or state to another, transition affects older individuals in many ways. In particular, relocation is a common transition for older adults and can be initiated by a change in health status, death of a spouse, decrease in financial resources, or the desire to be near family.

For people of any age, these changes are not only stressful and anxiety-ridden, but also a move to a different environment creates disequilibrium and chaos for an older adult when compared to younger counterparts [4,5,6,7]. However, many older adults are relocating to a more supportive living environment for multiple reasons, yet *how* they are able to achieve a positive transition into supervised housing is the focus of this integrative review. It will summarize, synthesize, and appraise research findings about older adults relocating from independent living to supervised housing using the Theory of Planned Behavior [8,9]. The aims that will be addressed in this review are: (a) to determine the behavioral beliefs of older adults who are relocating from independent living to supervised housing; (b) to determine the older adult’s level of perceived control of the relocation; and (c) to determine the influence of subjective norms on the older adult’s relocation.

### 1.1. Background

The U.S. Census Bureau reported that, from 2005 to 2010, 15.2% of adults age 65–74 and 11.9% of adults age 75 and older relocated [10] and moved within the same county of residence (8.5% and 7.1%, respectively) [10]. In 2013, 85% of adults aged 65 and older lived independently, some with spouses and some alone. That same year, 2.7% of older adults resided in senior housing and required assistance with at least one activity of daily living [2]. It is noted that the percentage of older Americans who enter a facility with 24-h supervision increases with advancing age [2].

Relocation is often a necessary process that older adults and their families often face as functional and cognitive declines necessitate environmental adaptations. Older adults who live alone or with family often lack adaptive amenities to compensate for functional declines (grab bars, safety rails, and emergency call system) while older adults living in communities designed for seniors were able to “age in place” for longer periods of time due to structural adaptations such as wheelchair access and safety bars [11,12]. Older adults are often compelled to move closer to adult children caregivers as the health of self or spouse deteriorates or merge into multigenerational households as do many African-American elders [13,14]. Therefore, the purpose of this integrative review is to determine the factors that facilitate positive transitions for older adults relocating from independent living to supervised housing.

Relocation has many implications for health care workers and families. Tang and Pickard [15] used secondary data from a previous study of community dwelling older adults and found that, the more services the respondents used (adult day centers, senior centers, housekeepers, visiting nurses, and personal aides), the higher the perceived need to relocate to a higher level of care [15]. Vulnerable older adults (*i.e.*, those with financial instability and low educational attainment) were not as adept in acknowledging the need for services or relocation as their functional status declined. Many independent older adults believe they will need to move in the future; however, their awareness and utilization of community services may be suboptimal [15]. Many relocation issues may be mitigated by improving awareness of available in-home or community-based services, such as adult day centers [5,15].

For many older adults and their families, the solution to increasing care needs is a relocation to senior housing. There are many iterations of supportive housing such as, assisted living, senior apartments, continuing care retirement communities, and naturally occurring retirement communities. Most all of these options provide security and maintenance free living, however, not all provide 24 h nursing supervision. Assisted living facilities are regulated by each state and as such, have widely variable staffing requirements and services but do provide room and board, assistance with activities of daily living, activities, and basic nursing care [16]. Currently, 735,000 older adults reside in assisted living facilities where the average length of stay is 22 months [17].

Bekhet, *et al.* [18] interviewed older adults who had relocated to senior housing (independent and assisted living). The results revealed multiple reasons for seeking a more supportive living environment.

“Pushing” factors toward a relocation were declining function, shedding home ownership responsibilities, lack of assistance, loneliness, and moving closer to family. “Pulling” factors reflected the older adults’ need for secure housing, reconnecting with friends, and familiarity with the senior living community. Some of the data revealed “overlapping” factors which were a combination of the “pushing” factors (needing to move) and the “pulling” factors (wanting to move). These findings have been substantiated in other research [7,19,20].

An acute care hospital stay and subsequent rehabilitation can significantly diminish the older adult’s functional status requiring a change in living arrangements [21]. Risk factors for relocation following a hospitalization are old age, being female, poverty, health problems, poor health behaviors, limited physical function, decreased hearing and/or visual acuity, and inadequate social support [22]. Older adults who reside by themselves, are located in a rural community, rent an apartment or house, and who cannot access support services may face relocation to a higher level of care after a hospital discharge [4,22].

For older couples, the reasons for relocation may include some of the issues previously mentioned (downsizing the family home, relocating nearer adult children, safety, *etc.*) but the timing of the move may be determined by the increasing frailty or declining health of one [18] spouse [5,23]. Additionally, lack of consistent, reliable caregiving help in the home, both formal and/or informal, often prompts a move to a different living situation [5].

### 1.2. Theoretical Framework and Definitions

The Theory of Planned Behavior (TPB) is an expansion of the Theory of Reasoned Action (TRA). The assumption of TRA is that intention to adopt a behavior is influenced by attitudes about performing the behavior (*behavioral beliefs*) and the value that important others (*referents*) place on the behavior (*subjective norms*) [8].

*Behavioral beliefs* are the perceptions that an individual develops concerning a specific behavior and are the antecedent to either positive or negative attitudes about that behavior while *subjective norms* are the opinions and beliefs that are communicated from others and the incentive to adopt those opinions about the behavior [24]. Perceived behavioral control was added to TRA to allow for the assumption that behavior is volitionally controlled and as such, is impacted by external factors that help or hinder performance of the behavior [24].

The following operational definitions were developed to simplify and standardize terms in this review. *Independent living* is defined as older adults living alone in their own home or apartment with minimal assistance from paid and/or unpaid caregivers while *supervised housing* is defined as older adults living in a congregate environment with 24-h supervision. Supervised housing is also described in the literature as congregate housing, assisted living, care homes, nursing homes, long term care, sheltered housing, and residential care [6,14,25,26,27].

Additionally, theoretical definitions are used to explicate the application of Theory of Planned Behavior concepts [8,24]. *Behavioral beliefs* are defined as the beliefs, perceptions, and experiences that the older adult considers when contemplating a move to a more supervised care environment. These beliefs entail reasons for the relocation, the impending move, and the decision-making process. *Perceived control* describes the level of perceived control the older adult has in decisions related to relocation. Perceived control manifests through the choices pertaining to relocation, reasons for relocation, and adjustment to a new living environment. Finally, *subjective norms* are the influence and opinions of referents (family, health care providers, and friends) on the relocation decision and transition to supervised housing.

The Theory of Planned Behavior has been widely used to understand behavioral intention to perform health behaviors and can be applied to both quantitative and qualitative inquiry [9,28]. Behavioral and control beliefs in addition to subjective norms, are identified through focus groups or interviews and enable the development of measurement instruments to quantify the beliefs most likely to encourage the health behavior of interest [28].

## 2. Experimental Section

### 2.1. Design

Articles were retrieved and assessed using a review style developed by Hawker, Payne, Kerr, Hardey, and Powell [29] (see Table 1 for criteria). This style has been utilized to evaluate qualitative and quantitative studies for systematic or integrative reviews [30]. The Hawker, *et al.* [29] method, also known as mixed research synthesis, allows for inclusion of research that measures concepts not well documented in traditional, clinically-based reviews [31].

Hawker *et al.* [29] advocates using a three-stage method for examining retrieved articles: Stage 1assess for relevance; Stage 2 for data extraction; and Stage 3-grade for methodological rigor. Stage 1 involves reviewing the articles resulting from the literature search. Articles are either accepted or rejected. Criteria for acceptance included applicability to the research question(s), appropriateness of the study particulars (sample, setting, researchers), origination of the data (professional or layman), and study methodology [29]. Stage 2 consisted of extrapolation of key data points about the studies to include design, sample, setting, research aim/hypotheses/questions, method and analysis, results, and conclusions. This step allows the reviewer to organize the major points of each study and further correlate the article with the review aims. Stage 3 entails evaluation of the articles using a four-category scoring mechanism based on specific criteria for components of the study. Ratings ranged from 1 (very poor) to 4 (good) and included the following study sections: abstract/title, introduction/aims, method/data, sampling, data analysis, ethics/bias, results, transferability/generalizability, implications/usefulness [29].

### 2.2. Method/Inclusion/Exclusion Criteria

Database searches were conducted using *relocation, transition, older adults, and elderly* as key words in the title and/or abstract of the manuscript. CINAHL, PubMED, and PsychInfo databases were searched for the years 1992 through 2014, with a return of 907 manuscripts. The search period was determined by the historical development and expansion of assisted living facilities in the United States [32]. Excluded from this review were dissertations, non-research based articles, books, and manuscripts that focused on physiological or biological transitions. Articles focusing on the relocation of older adults with dementia were excluded due to the variation in the cognitive abilities of these individuals that impact decision-making capabilities.

Once duplicates were removed and relevance assured, a total of 39 articles were reviewed for inclusion. Articles were reassessed for relevance of research on relocation transitions. Specifically, articles were included that addressed the following issues: behavioral beliefs about relocating to a higher level of care, perceived control over the decisional process, and the effect of the opinions of others (subjective norms). Reference lists were hand searched for those articles frequently cited in retained publications.

### 2.3. Synthesis of the Literature

Sixteen articles were retained for this review: Twelve were qualitative studies, three were mixed methods, and one was a quantitative design. Categories from Hawker [29] are used to organize the findings. (See Table 1 for criteria).

Abstract/Title. All abstracts contained complete yet concise information about the research, and all titles reflected the content contained in the publication.

Introduction/Aims. All articles except Fraher and Coffey [5] clearly provided an introduction to the problem and addressed the specific aims of the reported research.

Method/Data. All studies except Walker, Curry and Hogstel [23] used an appropriate methodology and data collection techniques. Walker’s examination of various dimensions of the relocation process might have been strengthened by using both quantitative and qualitative methodology to support the research aims.

Sampling. All articles had age-appropriate sampling from environments that addressed the transition experience. Most studies used convenience, opportunistic, or purposive sampling. One mixed methods study [33] used a random sample of participants from a larger study.

**Table 1:** Research assessment instrument [29].

Review Criteria	Good	Fair	Poor	Very Poor
**Introduction and aims:** Was there good background and clear statement of the aims of the research?	Full but concise background to discussion/study containing up-to-date literature review and highlighting gaps in the literature; clear statement of aim AND objectives including research questions	Some background and literature review/research questions outlined	Some background but no aim/objective/questions, OR Aims/objectives but inadequate background	No mention of aims/objectives
**Method and data:** Is the method appropriate and clearly explained?	Method is appropriate and described clearly. Clear details of the data collection and recording	Method appropriate, description could be better; data described	Questionable whether method is appropriate; method described inadequately; little description of data	No mention of method AND/OR method inappropriate AND/OR no details of data
**Sampling:** Was the sampling strategy appropriate to address aims?	Details (demographics) of who was studied and how they were recruited; why was this group targeted; the sample size was justified by the study; response rates shown and explained	Sample size justified; most information given but some missing	Sampling mentioned but few descriptive details	No details of the sample
**Data analysis:** Was the description of the data analysis sufficiently rigorous?	Clear description of how analysis was done: Qual. studiesdescription of how themes derived/respondent validation or triangulation; Quant. studies-Reasons for tests selected hypothesis driven/numbers add up/statistical significance discussed	Qual. studies-Descriptive discussion of analysis	Minimum details about analysis	No discussion about analysis
**Ethics and bias:** Have ethical issues been addressed and what has necessary ethical approval gained? Has the relationship between researchers and participants been adequately considered?	Ethics-Where necessary issues of confidentiality, sensitivity, and consent were addressed Bias-Researcher was reflexive and/or aware of own bias	Lip service was paid to (previous column)	Brief mention of issues	No mention of issues
**Results:** Is there a clear statement of findings?	Findings explicit, easy to understand, and in logical progression; tables, if present, are explained in text; results relate directly to aims; sufficient data are presented to support findings	Findings mentioned but more explanation could be given; data presented relate directly to results	Findings presented but haphazardly, not explained, and do not progress logically from results	Findings not mentioned or do not relate to aims
**Transferability or generalizability:** Are the findings of this study transferable (generalizable) to a wider population?	Context and setting of the study is described sufficiently to allow comparison with other contexts and settings, plus high score in (Sampling)	Some context and setting described, but more needed to replicate or compare the study with others, PLUS “fair” score or higher in (Sampling)	Minimal description of context/setting	No description of context/setting
**Implications and usefulness:** How important are these findings to policy and practice?	Contributes something new and/or different in terms of understanding/insight or perspective; suggests ideas for further research; suggests implications for policy and/or practice	Two of the (previous column) included	Only one of the (previous column) included	None of the above

Data analysis. Descriptions of data analysis were clear and understandable in all but three studies [5,34,35]. Wilson [34] and Armer [35] used a plethora of instruments and reported psychometric properties for all; however, no description of the statistical analysis performed on the results from the instruments was presented. Fraher and Coffey [5] did not describe their method for data analysis in their qualitative study. Wilson [34] discussed data analysis, but utilization of respondent verification of themes or triangulation was not included.

Ethics/Bias. Most reviewed studies reported institutional/university review board approvals, and some also reported approvals from facilities in which the studies were conducted. The majority of articles, qualitative in design, did not thoroughly address human subject protections or privacy issues. For example, Armer [35], Lee [36,37] and Leggett, *et al.* [38] did not describe the venues in which their interviews occurred, nor did they discuss how privacy was maintained for the participant. Johnson, *et al.* [39] did not report any human subject protections.

Results. Findings for all studies but Armer [35] clearly describe the outcomes with adequate supportive data and logical progression. Armer’s results section is very brief, lacks depth, and is primarily dominated by descriptive statistics and qualitative results. This brevity was surprising, especially considering the number of quantitative instruments described and utilized.

Transferability/generalizability. All studies report enough information for replication for further research. Since the majority of studies are qualitative, generalizability is not possible. However, reports of this research seek to describe behaviors, attitudes, and perceptions rather than quantify the process of transition experiences.

Implications/Usefulness. All studies offer suggestions for further research and processes for assisting older adults through a transition experience. Saunders and Heliker [19] advocate the establishment of an interdisciplinary team to assist new residents, families, and staff during the relocation transition process. Wilson [34] suggests a resident volunteer who would orient the older adult to his or her new home.

Using Hawker’s [29] process for examining the strength of the selected articles, six studies emerged that were inclusive of all the measurement criteria [18,19,27,33,40,41]. The abstracts and introductions impart to the reader a thorough synopsis of the study and a summary of supportive literature on the selected topic. The articles provide rich, detailed reporting of their methodology, sampling, data analysis, ethics, results, transferability, and implications. While the remaining articles [5,23,34,35,36,37,38,39] add to the general knowledge of relocation transition in older adults, a more comprehensive description of the introduction, method, data analysis, ethics, and results would have supplied the reader with an enhanced understanding of this important research.

## 3. Results

### Synthesis Summary

This section will summarize the results of the integrative review using the Theory of Planned Behavior concepts: behavioral beliefs, perceived control, and subjective norms. Articles were grouped into themed categories and explanations of how the definitions were addressed are included. (See Table 2 for Reviewed studies).

Behavioral beliefs are thoughts, perceptions, and idealizations of future actions or activities. For the older adult, perceptions about relocating to a supervised housing can influence the behaviors necessary to attain positive outcomes. In this research review, those attitudes can be classified into five categories: *perceptions of relocation, reasons for relocation, adjusting to relocation, decision-making, and “wild card”*.

Qualitative studies dominate the evaluation of older adults’ *perceptions of relocation* from independent living to supervised housing. Lee [37] interviewed independently-living elderly Chinese about their beliefs on residential care homes. Perceptions were mostly negative and described long term care as “a dumping ground” and “a place to die” [37]. The quality of care in congregate housing was also a concern for these older adults who felt that attentiveness of staff might not be adequate. Shippee’s [40] participants, who resided in a continuing care retirement community, viewed a move to a higher level of care as a disintegration of their established role as a healthy older adult, thereby disrupting friendships and social networks. Some older adults viewed relocation as a positive endeavor describing familiarity with the facility, social connections with current residents, closer proximity to adult children or other relatives, and, for those living alone, a means of combating loneliness [5,18,35,36,37,38,39].

*Reasons for relocating* to a higher level of care included physical and functional issues and psychosocial factors. The most common motivations were declines of the older adult’s or a spouse’s health and function [5,18,20,23,27,37,38,39]. Relinquishing home ownership and responsibilities, especially housekeeping duties for women, was another factor in relocating [18,36,38]. Inconsistencies in family support and in-home assistance, as well as unwillingness to move in with family, were additional reasons for older adults to relocate [18,37,38,39]. Interviews with African-American and European-American older adults revealed that indigent elderly may not be aware of or able to afford community-based care. Therefore, they had to move to a higher level of care sooner than their wealthier counterparts (Johnson, Popejoy & Radina, 2010) [33]. Several studies reported that fear of becoming a burden to families was a motivator for older adults who proactively relocated to a higher level of care [19,27,38].

*Adjusting to a new living environment* was reported in multiple studies and highlighted the differences in the belief systems held by older adults to navigate a new living situation. Some new residents experienced fear, anxiety, despondency, frustration, helplessness, anger, and stress [5,19,27,34,36,38]. These feelings subsided over time, but for those in whom the move was precipitous and unplanned, the adjustment period was longer [19,27,34]. However, an equal number of responses described relief of household duties, meeting new friends, improved safety, and increased socialization in addition to having continuous nursing supervision as benefits of relocating [5,19,27,34,36].

*Decision-making* throughout the relocation process involved the choice of when and where to move and also the loss of freedom to make decisions in a congregate living situation. Relocation decisions were usually made by the older adult, sometimes independently and sometimes with the assistance of family or health care providers [5,19,23,27,33,39]. In one study, residents of a continuing care retirement community expressed dismay that the decision to transition to a higher level of care would be made by a committee (resident director, nurse, facility administrator) after evaluation of the older adult’s functional abilities [40]. Autonomy was challenged by the rules, regulations, and absence of privacy at the new facility, lack of communication from staff, family and health care providers, and disruption of the older adult’s usual routines [5,34,36,37,40].

One study, “*wild card*”, defied classification within the parameters of the beliefs of older adults toward relocation. Walker, Curry and Hogstel [23] attempted to validate the physical and emotional toll (Relocation Stress Syndrome) experienced by elders transitioning from independent living to a supervised living situation. All the participants in this study expressed steadfastness and strength in the belief that moving was the best option for them and their families; this is not consistent with findings in the other studies.

Perceived control or the amount and quality of control the older adult has in the relocation decision-making process is directly related to positive outcomes and adjustment to a new living environment. The articles assessed for this section were classified into three categories: *reasons for relocation, decision-making, and adjustment to relocation.*

Older adults cited their own or a partner’s health and safety issues as the primary *reason to relocate* to supervised housing, and a decision they made either independently or with assistance from family or health care providers [5,18,19,20,23,33,34,38,39]. Other factors that contributed to a move were the desire to be closer to adult children, the desire for socialization, and elimination of household responsibilities. When the impetus to move was proactive or before an emergent event, older adults had more latitude in the decision-making process [18,20,23,39].

Armer [35] found that when a choice in relocation *decision-making* was perceived, the older adult experienced more positive outcomes during the transition. This finding was supported by Bekhet, *et al.* [41], Leggett, Davies, Hiskey and Erskine [38], and Walker and McNamara [20]. In contrast, elders who were unable or not allowed to have input into relocation decisions experienced prolonged adjustment periods, dissatisfaction with the facility and staff, and hesitancy to participate in the social activities of the community [27,33,34,40]. Participants in a continuing care retirement community who were assessed for self-care abilities by staff resented that they had no control over when the move would be deemed imminent [40]. Wilson [34] found that the majority of unplanned moves were a consequence of escalating functional decline and acute care hospitalization, resulting in a lack of decisional participation in relocating and a prolonged period of grief, anger, and regret.

*Adjustment to relocation* was a recurrent theme, particularly with loss of privacy, rules and regulations, and as well as coping with the loss of independence [19,27,34,35,36]. At the same time, some older adults expressed a renewed sense of safety, consistent care, and increased social interactions as a positive outcome to relocation [5,14,19,20,27,36]. Bekhet, Fouad and Zauszniewski [41] found that adjustment to relocation occurred more quickly if the older adult had participated in the decision to relocate. This finding was supported by Armer [35] and Leggett, Davies, Hiskey and Erskine [38]. V. Lee *et al.* (2013) interviewed older British adults and found that adjustment to relocation was not “time bound” but fluid and comprised of “plots” reflecting “control”, “power”, “identity”, and “uncertainty” [14]. Chinese elders’ acclimation to congregate living was mediated by cultural influences which enabled them to assume a new way of life and become one with their environment [36].

Subjective norms are the influences of “others” in changing perceptions and actions of behavioral change. Also termed “referents”, the approval, assistance, and guidance of these individuals was instrumental in assisting the older adult in the transition to supervised housing.

Family members were most often reported as assisting the older adult in the decision to relocate [5,35,38,39,40]. This varied from adult children, to extended family, or spouses. Health care providers, including nurses, social workers, and physicians, provided either support for the older adult’s decision to move or influenced them to pursue a higher level of care must be attained [27,33,39]. Relationship of the caregiver to the older adult was only revealed in qualitative narrative excerpts, limiting the ability to discern the type of caregiving affiliation.

**Table 2:** Reviewed studies.

Author	Aim/Purpose	Sample	Method	Key Findings/Themes
Bekhet, A., Zauszniewski, J. & Nakhla, W. (2009) [18]	To understand the reasons why elders move to retirement communities and what living in retirement communities is like from the perspective of relocated elders	Cognitively intact, relocated elders (*n* = 104) from a parent study	Qualitative interviews	Pushing factors; Pulling factors; Overlapping factors
Lee, V., Simpson, J., Froggatt, K. (2013) [14]	To explore qualitatively older people’s experiences after an initial adjustment phase in order to illuminate ongoing processes of transition and related psychological factors; to explore how transitions were internalized and reflected upon within residents’ life stories	Purposive sample; British older adults (*n* = 8)	Qualitative; interviews	Transition may be influenced by key plots of ‘uncertainty’, ‘identity’ and ‘power/control’, which are interwoven within individual’s daily and more long-term existence.
Walker, E., & McNamara, B. (2013) [20]	To identify key factors over different stages of relocation; to determine the range of strategies employed by older adults in relocating and maintaining a sense of home; to explore the scope for preventative occupational therapy in promoting health and well-being	Purposive/snowball sample; Australian older adults (*n* = 16)	Qualitative; semistructured interviews	Two main findings: successful transitions were made by researching and gathering information prior to the move and maintaining the ability to exercise agency across the relocation process
Johnson, R., Schwiebert, V., Rosenmann, P. (1994) [39]	To identify factors influencing placement of older adults in nursing homes; to delineate the process by which this decision occurred	Cognitively intact older adults (*n* = 18), >60 years, at least one year in nursing home, English speaking	Qualitative descriptive design; semistructured interviews; open ended questions	Factors Influencing placement: health issues, caregiver issues, fear of living alone; placement decision makers: “powerful other”/self; advice to others making placement decisions
Lee, D. (1997) [37]	To explore and investigate Chinese elder people’s perceptions of residential care placement	Convenience sample (*n* = 20); older adults >60 years from adult day centers	Quasi-qualitative; semi-structured taped interviews	Likelihood of residential care placement: Most (40%) believed a move would be necessary beliefs about residential care: Positive and negative perceptions Knowledge/experience of residential care homes: most information garnered from friends/acquaintances or volunteering in a care center
Lee, D. (1999) [36]	To achieve understanding of how Chinese elders in Hong Kong experience the changes associated with admission to residential care homes	Purposive sampling; older adults (*n* = 10) newly admitted to residential care homes; no hearing or speech deficits	Descriptive qualitative; audiotaped interviews within one week of admission	Positive and negative feelings about the move; Chinese culture encourages modifying expectations and adaptation; Communal living; Establishing new relationships
Shippee, T. (2009) [40]	To investigate how residents perceive transitions across levels of care and how residents manage social relations while moving within the CCRC	Purposive sample (*n* = 35)	Qualitative: observation and interviews	Autonomy; Threats to privacy/personal space; Fatalism; Social
Sviden, G., Wikstrom, B., Hjortsjo-Norberg, M. (2002) [27]	To describe the qualitatively different ways in which the participants said they experienced relocating to sheltered housing and adjusting to new living arrangements	Swedish older adults (*n* = 59) who resided in sheltered housing at least one year	Qualitative, exploratory, phenomenological approach; semistructured interviews	Reasons for moving to sheltered housing; Experiences related to reception at the sheltered housing; Adjustment to living in sheltered housing
Walker, C. , Curry, L., & Hogstel, M. (2007) [23]	To verify the nature and kind of distress associated with relocation stress syndrome (RSS); to validate diagnostic criteria for RSS among older adults residing in nursing homes and assisted living facilities; to determine whether RSS manifests differently among residents of one kind of facility *versus* another	Convenience sample (*n* = 16); nursing home (*n* = 8) and assisted living (*n* = 8) residents; >65 years, no greater than mild cognitive impairment	Qualitative; structured interviews	Moving from independent residence to LTC; Relocation differences between AL and LTC placement; Stressful relocation?
Wilson, S. (1997) [34]	To identify variance in the initial responses of older adults whose move into a nursing home is expected to be a permanent move and is either planned or unplanned	Older adults (*n* = 15) who had recently relocated	Exploratory, descriptive, qualitative interviews	Overwhelmed phase; Adjustment phase; Initial acceptance phase
Armer, J.(1993) [35]	To examine the relationship of perceived choice, perceived social support, cognitive appraisal, coping strategies, self-related health with adjustment to relocation of community based rural elderly	Residents (*n* = 50)of two congregate residential facilities; >60 years, without cognitive impairment or debilitating health conditions	Mixed methods: Cross-sectional, descriptive correlational; semi-structured interviews; Questionnaire with instruments	Instrument scores correlated significantly with perceived choice in relocation and current environment, social support (family/neighbors), predictability, threat appraisal, challenge appraisal.
				Qualitative themes: Most reported positive feelings toward relocation; Perceived choice in relocation/environment improved adjustment
Johnson, R., Popejoy, L., Radina, E. (2010) [33]	To identify extent of older adults’ participation in relocation decision making and extent of SOC (sense of coherence), function, physical ability as related to decision making.	Random selection of nursing home residents (*n* = 16)	Mixed methods: qualitative interviews and four instruments:	Qualitative: Two themes: “They put me here” & “I made the decision.”
				Quantitative: Significance was not attained
Leggett, S., Davies, S., Hiskey, S., & Erskine, J. (2011) [38]	To explore the application of the Time, Environment, Motivation, Personality, and Outcome (TEMPO) model and establish whether an increase in frequency of prefactuals/counterfactuals might emerge as people move along the TEMPO timeline	Opportunistic sampling (*n* = 66) divided into two groups: Age 18-64 (*n* = 33) and age >65 (*n* = 33)	Mixed methods; cross-sectional; Interviews using two fictitious scenarios about relocation	Qualitative-multiple themes focused on preplanning for a move due to poor health
				Quantitative-Older adults tended to view scenarios as opportunities to plan ahead
Bekhet, A., Fouad, R., & Zauszniewski, J. (2010) [41]	To determine whether the effects of risk factors (relocation) on elders’ resilience (adjustment) are influenced by protective factors such as positive cognitions	Convenience sample of Egyptian older adults (*n* = 94) who had relocated to retirement communities	Cross-sectional; quantitative; three instruments	Mediation: Relocation controllability had a direct negative effect on relocation adjustment (*B* = −0.36, *p* < 0.001)
				Relocation controllability had a direct negative effect on positive cognitions (*B* = −0.41, *p* < 0.001)
				Effect of relocation controllability and positive cognitions on relocation adjustment (*B* = −0.20, *p* < 0.05)

## 4. Discussion

Supporting the Theory of Planned Behavior, behavioral beliefs and perceived control are well-documented in the articles reviewed. Studies reflect the duality of the older persons’ beliefs and perceptions of control over their destiny. For some, transition into a new living environment was welcomed because it decreased the burden of home ownership, provided a safer and more secure living situation, and afforded continuous supervision and nursing care. Other older adults, especially those whose relocation was forced or unexpected, suffered from emotional distress, loss of self-worth, and inability to integrate into the new community. For them, the move did not provide the same level of acceptance or positive outcomes. The majority of participants, whether willing or unwilling movers, experienced some amount of sadness and grief over the loss of independence, but most were eventually able to accept their physical declines and increased care needs. An interesting finding indicated that some older adults had difficulty “looking” at their own futures embodied by fellow residents who were more infirm and needy [5,27,36,40]. This realization occurred sometime after admission and limited socialization and participation in activities. Manifestations of both positive and negative transitions to a higher level of care are described in these studies and reflect an increased awareness of the power and magnitude of behavioral beliefs and perceived control on positive transitional experiences.

Those who assist the older adult in the relocation decision process (referents) are viewed in the studies as one entity. Some of the qualitative narrative identifies a specific relationship (daughter, niece) or profession (physician, social worker) but most referents are termed “family”. Although the literature presented here heralds and supports the role of family, the term is a misnomer. Family caregivers can be family, friends, partners, and neighbors, but the most common caregiver is a 52 year old female caring for her widowed mother [42]. Filial caregivers are the adult children of older adults who assume the caregiver role for one or more parents while caring for dependent children and maintaining full time employment. Sandberg, *et al.* [43] examined the role of the adult child in the transition of one parent to a higher level of care while still supporting the “healthier” parent. The degree of involvement of the adult child was determined by the needs of the remaining parent. The adult child influenced the decision to move the sicker parent and remained an active participant in caregiving for both parents. Additionally, the adult child assumed the burden of guilt associated with the placement decision. After relocation, the adult child’s caregiving role is revised to incorporate maintaining contact with life outside the care facility and monitoring care received by paid staff [44]. Future research should explore the role of the family caregiver by clearly defining the relationship to the older adult and the depth of their involvement in the caregiving and relocation processes.

Another area of research should focus on how the Baby Boomers are planning for their long term care needs, many of whom are caring for aging parents. Robison, *et al.* [45] surveyed randomly selected Baby Boomers to determine their engagement in long-term care planning. Although 60.1% of the respondents felt they would require assistance as they aged, 77.4% of Baby Boomers had no plans for financing future care needs or did not know how they would pay for care as they aged [45]. This is particularly disturbing in that this cohort will significantly swell the aging population and tax an already fragile system of social supports for older adults.

### Limitations

This review was time-limited to literature published from 1992 to 2014. Other publications released prior to 1992 may have provided a different historical perspective on relocation of older adults as they would focus on a different generational cohort. Additionally, studies that involved the relocation of older adults with dementia were excluded. While this phenomenon is widely researched, it would be difficult to fully explicate the impact of perceived control, behavioral beliefs, or subjective norms in older adults whose communication and recall may be impaired. Some of the studies were multi-cultural (African American, Egyptian, Chinese, Scandinavian); however, only one addressed the issue of relocation experiences for low-income older adults [33].

## 5. Conclusions

The majority of studies for this integrative review are qualitative, and the few that are quantitative used a multitude of instruments to determine the elements of a relocation transition for older adults. It is interesting that all of the studies essentially arrive at the same conclusion: Positive relocation transitions are based on the older adults’ belief that a move is necessary and that there must be some level of choice and control over the decisions made prior to the relocation. This finding reflects the tenets of the Theory of Planned Behavior in that older adults believe they will need to relocate and when possible, should have control over the decisions surrounding a relocation. While some older adults make these decisions independently, many rely on others to assist in the process of making a move.

How then, do health care professionals facilitate these two vital components so that older adults can transition to supervised care with minimal emotional turmoil? The answers, gleaned from the research evaluated here, are to explore the older adult’s preferences for supervised housing and to encourage proactive decision-making about alternate housing options. Open, consistent, and honest conversations with older adults and their families are essential to avoid the poor outcomes that result from an emergent relocation. Other avenues of research should focus on methods to mitigate or alleviate the major issues expressed by the participants, such as lack of privacy, inflexible rules and regulations, and suppression of autonomy.
